# Recent developments in the funding and organisation of the New Zealand health system

**DOI:** 10.1186/1743-8462-2-9

**Published:** 2005-05-07

**Authors:** Toni Ashton

**Affiliations:** 1 Centre for Health Services Research and Policy, School of Population Health. University of Auckland, Private Bag 92019, Auckland, New Zealand

## Abstract

During the 1990s, the New Zealand health sector went through a decade of turbulence with a series of major structural changes being introduced in a relatively short period of time. The new millennium brought further change, with the establishment of 21 district health boards and the restoration of a less commercially-oriented system. The sector now appears to be more stable. However many incremental changes are in train and there has been considerable turbulence below the surface as key players jostle for position. This paper reports on some of the recent changes that have occurred in the restructuring of the New Zealand health system. Three issues are discussed: the devolution of funds and decision-making to district health boards, developments in primary health care, and the position of the private health insurance industry.

## Introduction

The New Zealand health system has gone through a series of fairly radical structural changes over the past decade or so. Most notable – and most radical – were the 1993 so-called "health reforms" in which an attempt was made to introduce market-like incentives into the system by requiring public and private providers to compete for service contracts from public purchasers. Although it was probably too early to expect any major improvements in health sector performance, the general consensus that emerged from policy analysts was that the new system was unlikely to achieve any significant efficiency gains [1-3]. The competitive arrangements were also rather alien to many of those working within the health system who were more accustomed to a public sector style of philosophy [4].

The new millennium brought yet another round of restructuring following the election of a Labour-led coalition government. The general direction of change this time around was towards a more planned and community-oriented system, with 21 district health boards (DHBs) being responsible for meeting the health and disability service needs of the people living within their district. The aims of the restructuring into DHBs are set out in the New Zealand Public Health and Disability Act 2000. In addition to providing appropriate health and disability services for all New Zealanders, the objectives are to reduce health disparities (especially by improving the health outcomes of Mäori); to foster community participation (via elected representatives, openness of decision-making and community consultation on strategic planning); and to facilitate access to and dissemination of information pertaining to service delivery.

A strong primary care system is seen as central to improving the health status of the population generally and, more particularly, to reducing health inequalities. The government has therefore given priority to implementing the Primary Health Care Strategy [5]. The strategy is complex and multi-pronged, and includes changes to both the organisation of primary health services and to the level and method of subsidising these services.

As in 1993, the fundamental method of financing health care through general taxation has remained unchanged during this latest round of reform. While the share of public spending in total health expenditure declined from a high of 88.1% in 1981/82 to 76.6% in 1992/93, it has since remained fairly stable at around 77% of total health expenditure [6,7]. However, after increasing steadily from 2.8% of total health expenditure in 1989/90 to 6.8% in 1996/97, the share of private health insurance in total funding subsequently declined to 5.7% in 2001/02 following a series of rises in premiums [6,8]. The lack of growth in membership of private health insurance, together with the subsidisation of private insurance in Australia, has encouraged the health insurance industry to lobby the government for a greater role for private insurance in financing the health system.

We are now three years into the new structure. The DHBs are well-established, with the second round of elections for board membership having taken place in October 2004. DHBs have been active in working with primary health care providers to establish networks of providers called Primary Health Organisations (PHOs) and by April 2005, 77 PHOs had been established covering more than 90% of the population [9]. While significant progress has been made away from the commercially-oriented environment that prevailed during the 1990s towards a more community-focussed health system, the path has not always been smooth. This paper describes and discusses three particular issues: the process of devolving funds and decision-making to the DHBs, developments in the primary health sector, and the private health insurance lobby. To place the issues into a historical and political context, the paper begins with a brief overview of the New Zealand health system and its development. Each of the three issues is then described and discussed in more detail.

## Overview of the New Zealand health system

The roots of the New Zealand health system as it is today were first formed through the Social Security Act 1938 when the (Labour) government of the day outlined its vision of free health services for all New Zealanders, regardless of ability to pay. However widespread opposition from the medical profession meant that the Act was never implemented in full [2,10]. Instead, a dual system of funding emerged in which mental health, maternity, and hospital services were fully funded by the government, while GPs retained the right to charge a fee over and above any subsidy for general practice consultations. A dual system of provision also emerged, with most primary services being provided in the private sector but most secondary and tertiary services being provided by public hospitals. As public provision of hospital services expanded, the number of private hospital beds initially declined. However, the introduction of private medical insurance in the early 1960s, together with subsidies for the maintenance of private hospitals, eventually reversed this trend. [10,11].

By the 1970s, concern was growing that "the fragmented pattern of health care delivery means that New Zealand lacks a national health service" [12]. After a series of reviews and proposals, 30 local hospital boards were eventually gradually replaced by 14 area health boards between 1983 and 1989. The funding and provision of public health services and public hospital services were amalgamated under area health boards. However, primary medical services remained separately funded and provided. Thus the vision of a "national health service" remained illusive.

Area health boards had a number of features that were subsequently reintroduced in 2001 as part of the district health board structure. These features include:

• governance by a locally-based and (mostly) locally-elected board;

• funding by means of a population-based formula;

• a reorientation away from curative services towards prevention;

• planning of services in consultation with key stakeholders;

• a more strategic approach to health service delivery, including the use of national goals and targets.

Establishment of the 14 area health boards had not long been completed before they were abolished in 1991 and the so-called 'purchaser-provider split' was introduced in 1993 following two-years of preparation. In reality the split only applied to those services that had previously been provided by the area health boards: that is, public hospital services, public health services, and a limited range of community-based services. The roles had always been separated for most primary health services and no attempt was made to apply the principle to privately-funded hospital services. The main objective of separating the roles of purchaser and provider was to secure efficiency gains by introducing market-like incentives into the health system and requiring a more commercial approach to health service delivery. The separation also effectively allowed, for the first time, funding for all personal health services (i.e. including primary health services) to be amalgamated into a single funding stream. Funding for public health services was initially "unbundled" and given to a separate purchasing agent so as to ensure that these funds could not be spent instead on treatment services. However, these arrangements were short-lived and from 1996 the same purchasing authorities became responsible for both personal and public health services (although funding for public health services remained ear-marked specifically for this purpose). Between 1991 and 1997, the formal locus of responsibility for purchasing health services shifted from local purchasing (under 14 area health boards), to regional purchasing (under 4 regional health authorities) to central purchasing (under a single health funding authority) [13].

The nature and impact of these changes have been discussed in detail elsewhere [1,3,10,13]. Of relevance for this paper is the fact that, while funding for primary health care had effectively been merged with the funding of other services, New Zealand still lacked a coordinated national health system. The system was unplanned and often uncoordinated, service delivery was still fragmented, and problems of access to primary medical care due to high patient copayments remained. In addition, the Labour party, which won the right to lead a coalition government following the 1999 election, was ideologically opposed to "a model which promotes competitive tendering for contracts" [[14], p4].

Restructuring commenced once again early in 2000, the key aim being "the restoration of a non commercial system, with the focus on the provision of quality services" [[14], p.4]. Responsibility for purchasing services was transferred from the central purchasing agent (the Health Funding Authority) to the Ministry of Health as a temporary measure until the 21 DHBs could be established. As noted above, DHBs have a number of parallels with area health boards. However the range of services covered by DHBs is wider than those covered by area health boards. Most significantly, their responsibilities include primary health services as well as secondary and tertiary care. Some of the issues that have arisen during the process of devolving funds and decision-making to the DHBs are discussed in the next section.

## Devolution of funds and decision-making to district health boards

Between 1993 and 1999 when the purchaser-provider split arrangements were in place, health services were purchased from providers largely on a cost-and-volume basis. A return to population-based funding at the district level therefore requires some redistribution of funds across the 21 districts. The formula that is now being used for determining a district's share of public funding takes into account the demographic make-up of each district, plus additional adjustments for unmet need, overseas visitors and the degree of rurality. Perceived problems with the population-based funding formula mean that many DHBs remain unhappy with their resultant quotas. These perceived problems include: systematically inaccurate population forecasts (primarily as a result of rapid internal migration); inadequate adjustment for people who have a high need for services but who historically may have under-utilised these services; no adjustment for new immigrants who often have special health service needs; and the possibility of 'medical migration' of people with on-going special service needs to the larger boards which are able to provide a more comprehensive service. There has also been much debate over the speed with which funds should be reallocated away from those boards which are over-funded to those that are under-funded. These issues have created some tensions amongst the DHBs. In the longer term, the aim of achieving equitable access to services for all New Zealanders may continue to be compromised if there are inherent inequities within the funding formula.

The initial establishment of the 21 DHBs reportedly went relatively smoothly [15]. In part, this was because, in an effort to minimise disruption, the number and size of the DHBs were configured to match the services and implicit boundaries of the publicly-owned organisations (previously called Hospital and Health Services). This pragmatic approach facilitated implementation of the new model and kept the associated costs down. However it also created a rather large number of boards for a population of only four million. A large number of DHBs increases transaction costs (for example, of the associated bureaucracy and of tracking the flows of patients across district boundaries) and may result in losses of potential economies of scale. It has also meant that scarce expertise is spread very thinly, especially in areas such as Mäori health and public health [[15], p.100]. The size of the 21 DHBs varies between about 33,000 and over 430,000 people [16]. The dynamics, management and issues facing these 21 organisations therefore vary significantly. The aim of the government to achieve equity of access to services for all New Zealanders in all regions may be difficult where there is such diversity amongst those organisations which are responsible for allocating the funds to providers within their districts.

While DHB establishment was itself relatively smooth, the process of devolving funds and decision-making to the districts has not been trouble-free. Devolution of funds involved, amongst other things, the transfer of responsibility for numerous contracts for services from the Ministry of Health to the DHBs. The DHBs found that many of the contracts were either inaccurate or incomplete and in some cases there were long delays in getting copies of the contracts [15]. The DHBs therefore did not have the information that they required to monitor the services being provided under the contracts.

Devolution of the funds for some services has also not occurred as soon as had originally been envisaged. By early 2005, the Ministry of Health still retained responsibility for the funding of all public health services, and for disability support services for people aged below 65 years. The ministry has also retained control over much discretionary spending, so that new money transferred to DHBs is sometimes already tagged for spending on specific services. Reasons for this lack of full devolution of funds are unclear. It may be associated with a limited capacity in some DHBs to manage more contracts. More fundamentally, it may reflect a perception on the part of the ministry that, notwithstanding the desire to encourage responsiveness to local needs and preferences, the purchasing of some services – particularly some public health services – may be better managed at the national level.

Even when funds *have *been fully devolved to the DHBs, this does not imply full autonomy by DHBs in decision-making. A key feature of the reforms this time around has been the development by the Ministry of Health of two overarching national strategies to provide strategic direction for the health and disability sectors as a whole and to ensure a degree of national consistency where decisions are decentralised [17,18]. Strategies have also been drawn up to guide the development of services for sub-groups of the population (such as Mäori and older people) [19,20] and for various sectors of the health system (such as primary health care) [5]. The 21 DHBs are required to adhere to the framework and priorities outlined in these national strategies when drawing up their annual plans and strategic plans, all of which must be signed off by the Minister of Health. However the boundaries between the responsibilities of the Ministry and of the DHBs are, as yet, by no means clear. While the DHB model shifts the locus of decision-making for the funding and provision or purchase of services to the district level, control over strategic direction by the central government constrains local decision-making. Moreover local and national priorities may sometimes be in conflict.

The minister has sometimes shown some reluctance to allow DHBs to make decisions about the provision of particular services in their areas, especially where this involves some disinvestment in services. For example, on one occasion the minister reversed a DHB's decision to stop providing after-hours surgical services in a rural hospital because of concerns about patient safety. This is seen by some DHBs as the minister interfering with local preferences [[15], p.98]. As the DHBs become more established and more experienced, the minister and the ministry may be more willing to allow the DHBs a greater degree of autonomy in decision-making. However, these tensions between the centre and the regions are by no means unique (or new) to New Zealand. Rather they reflect the hierarchical nature of a tax-funded system in which one organisation (the central government) is responsible for financing the system while other organisations (the DHBs) are responsible for spending the money.

## Developments in the primary health sector

As noted above, recent developments in the primary health sector include changes both in the way that services are organised as well as in the method and level of subsidy for these services. The restructuring involves the grouping of general practitioners (GPs), primary care nurses and other primary health care providers under umbrella groups called Primary Health Organisations (PHOs). These PHOs are non-profit organisations which contract with DHBs to provide a comprehensive set of preventive and treatment services for their enrolled populations. PHOs are required to involve their communities in their governing processes, and to work with their enrolees to develop services that reflect their particular priorities and needs. In some districts, PHOs have been established on a geographic basis so that membership is determined by area of residence. In other districts, people can choose between two or more PHOs so that PHOs effectively compete both for GPs and for individual patients. PHOs – like DHBs – vary in size, from around 3,000 patients to over 330,000 [9].

With respect to the funding of primary health care, a key goal of the government's Primary Health Care Strategy is to remove the cost barrier that currently deters some people from seeking care. Government subsidies for general practice services (and also for pharmaceuticals) have historically been paid on a fee-for-service basis in New Zealand, with subsidies being targeted to low income and high risk people. Because the subsidy levels have not been regularly increased with inflation, and because GPs have retained the right to set their own levels of copayments, this has resulted in a significant cost barrier for some people to GP services [21,22]. In a national survey undertaken in 2002/03, around 6% of adults reported that they had not visited a GP within the last 12 months because of cost [22].

In an effort to remove or reduce this cost barrier, the move to PHOs is being accompanied by three changes to the way that government subsidies for primary care are paid:

• a change in the way that the general medical service subsidy is paid from fee-for-service payments to GPs to capitation funding of PHOs;

• the phased introduction of higher government subsidies for general practice services and pharmaceuticals for all New Zealanders [23];

• a shift from subsidies that are targeted towards high need individuals towards subsidies that are paid on a universal basis.

The bulk of government funding paid to PHOs is determined by two alternative capitation formulas, one of which – the 'Access' formula – provides a higher rate of subsidy. This formula applies only to those PHOs in which 50% or more of their enrolled population is either Mäori or Pacific, or living in a deprived area (as defined by the NZDep2001 Index which combines 8 census variables that reflect aspects of social and material deprivation.) All other PHOs are paid under the 'Interim' formula at a lower capitation rate. As the name suggests, payment to providers at this lower level is intended only as an interim measure, the intention being to gradually extend the higher rate of subsidy to all New Zealanders over the next few years. Higher rates of subsidy are now being paid to all PHOs for children up to the age of 17 years (since October 2003) and for those aged 65 years or over (since July 2004). Subsidies will be gradually increased for other age groups through until July 2007, at which time the higher capitation rates will apply to the whole population [23].

Payment to PHOs by the two different formulas has introduced some inequities into the system and caused considerable concern amongst many people working within the sector. Because the capitation payment covers all enrolees in a PHO, wealthier people who belong to PHOs that are paid under the Access formula will be paying less for GP consultations than poorer people who belong to other PHOs.

The differential in the subsidy levels has also stimulated aggressive – and sometimes acrimonious – competition between providers in some districts. It has encouraged PHOs to compete to enlist general practices that have a high proportion of deprived people on their registers. It has also encouraged individual practices to actively enrol particular patients as a means of obtaining eligibility for higher subsidies through the Access formula [24]. In those areas where there are both access and interim PHOs, patients have an incentive to shop around amongst GPs on the basis of price. From an administrative perspective, the move from fee-for-service reimbursements to capitation payment has meant that PHOs have had to tackle many technical difficulties and set up new management systems during the establishment phase [25]. All of these pressures have created a rather unstable environment which does not align with the government's vision of a primary health sector in which services are specifically tailored to meet the needs of a stable and identifiable population.

Preliminary evaluation of the impact of the subsidy increases on patient fees indicates that the fees being charged to patients by GPs have not always fallen by as much as might have been expected, had the subsidy increases been passed on in full to patients [26]. A survey of GP fees in February 2004 showed that GPs belonging to PHOs funded by the Access formula were generally charging all of their patients fees that are significantly lower than other PHOs [27]. However fees in other PHOs (i.e. those paid under the "Interim" formula) were generally higher than in GP practices which did not belong to a PHO at all. This indicates that the higher subsidy paid to PHOs has not always been passed on to patients as the government had hoped. A later survey found that the fees charged to people aged 65 and over fell by an average of 24% following the introduction of a patient subsidy for this group on 1 July 2004 [26]. However fees charged to these patients had *increased *by an average of 12% in the months prior to the introduction of the subsidy.

As a result of the information from this evaluation, the government is now working more closely with PHOs and District Health Boards in an effort to ensure that GP fees are set at reasonable levels. However, subsidy levels are not automatically adjusted in line with inflation. As long as GPs retain the right to set their own fee levels, universal low cost access to primary health care could prove difficult to achieve and to sustain in the longer term.

## Private health insurance

Unlike Australia, private health insurance in New Zealand is unregulated and, since the abolition of the tax deductibility of premiums in the late 1980s, does not receive any direct financial assistance from the government. It has also not been a topic of any significant public debate. However, the issue is of interest, first, because government policy towards private insurance industry in New Zealand contrasts sharply with that in Australia, and second, because the industry is currently lobbying for change based upon much the same arguments as those that were used to support the introduction of the Private Health Insurance Incentive Scheme in Australia.

In October 2004, the insurance industry published two reports within two days, both of which lobbied for direct government assistance to private insurance as a means of enhancing efficiency, equity and choice within the health sector [28,29]. The first report was commissioned by the Southern Cross Medical Care Society, which provides health insurance to around two thirds of the people who have private health insurance cover in New Zealand. Written in collaboration with some Australians, the report claimed that the health insurance industry in New Zealand faces "serious decline" and that, without government assistance, health insurance coverage "may halve over the next 10 years" [[28], p.i]. The proposed solution was a 30% rebate on insurance premiums akin to that in Australia. The authors argued that the Australian experience ".....shows that a rebate on health insurance premiums has boosted coverage to a healthy level, reduced pressure on the public health system, improved the fairness of the health system (by the government paying some health costs of both insured and uninsured people) and generally secured the future of the health insurance industry" [[28], p.i].

The second report, published by the Health Funds Association of New Zealand (i.e. the body that represents the interests of the health insurance industry), claimed that "public health inflation is at record levels" [[29], p.1] and that such rates of increase "will quickly become unsustainable" [[29], p.13]. Based upon an estimated public health inflation factor of 8% per annum over the last 3 years, the report projected that public health expenditure would reach 63% of GDP by 2050! The report went on to suggest that costs could be contained if contestable funding was to be introduced into the DHB system. It proposed that people earning in excess of NZ$38,000 should be required to purchase their own health insurance, with the government refunding the amount of their tax that would otherwise have been used to purchase health services. Any contributions made by employers should be exempt from the fringe benefit tax. DHBs would then sell their services to those people who are privately insured at a price equal to the true cost of the service. The report claimed that such a system would encourage both DHBs and individuals (or their employers) to focus on value for money, thereby providing the necessary incentives to keep health inflation down.

Interpretation of the data which form the basis for the claims made in each of these reports is highly questionable. This comment applies both to the evidence presented to illustrate that there is a problem with current financing arrangements in New Zealand, as well as to the impact of the proposed solutions. Even the basic premise that private health insurance is in serious decline is not well supported. While the proportion of the population covered by private insurance has indeed declined from a peak of around 45% in the late 1980s [30] it has remained fairly stable at around one third of the population over the past five years [28]. And although the proportion of insured with comprehensive cover (as opposed to cover mainly for hospital services) has declined from 20% in 2000 to 14% in 2004 [28], this could equally reflect improvements in access to publicly-funded primary health services as much as a response to increases in insurance premiums.

It is also difficult to accept that New Zealand should follow Australia in introducing a 30% tax rebate on premiums. In Australia, the rebate was one part of a package of subsidies and regulations. Separating out the precise effects of the rebate from the effects of the other components of the package is problematic and requires the adoption of a number of assumptions. Even so, there appears to be some consensus amongst analysts that, while the rebate does appear to have stimulated an increase in insurance coverage in the short term, the size of the effect may have been less than the government expected, and may not have been large enough to justify the expenditure [31,32]. Moreover, recent figures suggest that health insurance coverage in Australia is now declining [33].

It is even more difficult to justify a subsidy on the grounds that it will reduce pressure on the public health system. As Richardson recently pointed out in this journal, in Australia, changes in public and private bed numbers indicate that problems of access to the public health system are determined primarily by constraints on the supply side, rather than by an excess demand caused by an inability to afford private health insurance [34]. Vaithianathan, too, has shown that the demand for public hospital beds is unlikely to decline because an insurance subsidy is most likely to increase insurance coverage of people who previously paid directly for the use of private hospital beds, rather than of people who currently use public hospitals [35]. Even if a subsidy does actually encourage a shift from the public sector to the private sector, Frech III and Hopkins have suggested that, from a theoretical perspective, the optimal subsidy may actually be negative (i.e private health insurance should be taxed) [32].

In the second report [29], while the main justification for a greater role for private health insurance was escalating costs in the public sector, the meaning of the term "public health inflation" was unclear. In some instances [e.g. [29], p.12], the term seems to apply to changes in public health expenditure, while in other cases it apparently refers to increases in public hospital costs adjusted for hospital throughput [[29], p.9]. Neither of these are good indicators of cost increases across all of the services that are publicly funded, but either way, the estimated figure appears to have been simply extrapolated to the year 2050, thus producing an estimate of public health expenditure that is both excessive and, more importantly, newsworthy. Even if such a figure could be substantiated, there is little evidence from the international literature in support of the claim that contestable insurance funding is likely to assist in controlling costs. If anything, total health expenditure tends to be higher in insurance-funded systems than in systems that are predominantly funded by general taxation [36,37]. Reasons for this higher expenditure include the difficulty of containing costs in a system where there are multiple purchasers, and where reimbursements are usually on a fee-for-service basis.

While the government did not respond publicly to the claims and proposals made by the health insurance industry in these two reports, it did take two decisive actions. First, it requested briefings from the Treasury on both of the reports immediately prior to their public release [38,39]. Second, on the same day that the second report was published the Ministry of Health released its own report on the future funding of health services in New Zealand [40]. One of the conclusions from this report – which had in fact been written for the Ministry two years earlier but which had not been released – was that "there should be no public subsidies of private health insurance in New Zealand" [[40], p.xiv]. The main reasons behind this conclusion were (a) inequalities are likely to be exacerbated, because expenditure on private insurance increases with income; (b) control over health expenditure is more difficult under private insurance than under direct public funding; (c) greater value is likely to be achieved by increasing expenditure in the public sector because service provision tends to be more expensive and administration costs tend to be higher in the private sector; and (d) because demand for private insurance is relatively insensitive to price changes, the cost of a health insurance subsidy will be greater than the value of any health services that are stimulated by that subsidy.

In summary, while a rebate on private insurance may well improve the health of the private insurance industry, there appears to be little evidence that it would make any useful contribution towards improving the health of New Zealanders. More fundamentally, the current government would require any changes to financing arrangements to align with the principles which underpin the New Zealand Health Strategy. One of these principles is: "timely and equitable access for all New Zealanders to a comprehensive range of health and disability services, regardless of ability to pay" [[17], p.vi]. As Richardson has noted, the egalitarian desire of equalising access to health care regardless of ability to pay, and reducing inequalities in health are "more easily achieved through a compulsory public health system" [[34], p.5]. In contrast, contestable funding and subsidisation of private insurance are more appropriate for a health system aimed at maximising individual choice.

## Conclusion

This paper has described and discussed just some of the issues that currently face the New Zealand health system. Many of these issues are not new but rather are renewed manifestations of debates which have been recurrent features in various waves of health sector reform. In particular, the division of responsibilities between the centre and the regions, uncontrolled copayments for GP consultations, and the tax treatment of private health insurance are all issues which have repeatedly challenged decision-makers.

During any period of change, there are inevitably conflicts, tensions and disagreements as new boundaries are drawn and the various players jostle for position. This certainly has been, and continues to be, the case in this latest round of reforms in New Zealand, most particularly in the primary health care sector. As the system matures, the boundaries of responsibility across the sector should become clearer. If the vision of a community-oriented system is to become a reality, full devolution of responsibilities from the centre to the districts will be essential as the DHBs continue to build their capacity, capability and experience. The Ministry of Health can then focus on providing strategic direction to the sector and on monitoring performance through appropriate accountability mechanisms.

In spite of the tensions and difficulties associated with implementation of the new structure, the sector already appears to be developing a much stronger sense of direction and purpose. This is in sharp contrast to the 1990s when the change towards a more market-oriented system resulted in a high degree of uncertainty for, and alienation of, many service providers [41]. Another difference is that the government has invested in a number of evaluation projects by health service researchers this time around. These evaluations should highlight the main strengths and weaknesses of the new institutional arrangements, and, where necessary, indicate areas where further adjustments are required.

## Competing interests

The author(s) declare that they have no competing interests.
